# A risk model based on 10 ferroptosis regulators and markers established by LASSO-regularized linear Cox regression has a good prognostic value for ovarian cancer patients

**DOI:** 10.1186/s13000-023-01414-9

**Published:** 2024-01-04

**Authors:** Tingchuan Xiong, Yinghong Wang, Changjun Zhu

**Affiliations:** 1 Department of Gynecologic Surgery, The 3rd Affiliated Teaching Hospital of Xinjiang Medical University (Affiliated Cancer Hospital), Urumqi, 830011 China; 2 Center of Heath Management, The First Affiliated Hospital of Xinjiang Medical University, Urumqi, 830011 China; 3 Tianjin Key Laboratory of Animal and Plant Resistance, College of Life Sciences, Tianjin Normal University, Tianjin, 300387 China; 4 Laboratory of Molecular and Cellular Systems Biology, College of Life Science, Tianjin Normal University, Tianjin, 300387 China

**Keywords:** Ovarian cancer, ESTIMATE algorithm, Immune score, 10-ferroptosis regulator and marker signature, LASSO-regularized linear Cox regression

## Abstract

Ovarian cancer is the deadliest gynecologic cancer due to its high rate of recurrence and limited early diagnosis. For certain patients, particularly those with recurring disorders, standard treatment alone is insufficient in the majority of cases. Ferroptosis, an iron- and ROS (reactive oxygen species)-reliant cell death, plays a vital role in the occurrence of ovarian cancer. Herein, subjects from TCGA-OV were calculated for immune scores using the ESTIMATE algorithm and assigned into high- (*N* = 185) or low-immune (*N* = 193) score groups; 259 ferroptosis regulators and markers were analyzed for expression, and 64 were significantly differentially expressed between two groups. These 64 differentially expressed genes were applied for LASSO-regularized linear Cox regression for establishing ferroptosis regulators and a markers-based risk model, and a 10-gene signature was established. The ROC curve indicated that the risk score-based curve showed satisfactory predictive efficiency. Univariate and multivariate Cox risk regression analyses showed that age and risk score were risk factors for ovarian cancer patients’ overall survival; patients in the high-risk score group obtained lower immune scores. The Nomogram analysis indicated that the model has a good prognostic performance. GO functional enrichment annotation confirmed again the involvement of these 10 genes in ferroptosis and immune activities. TIMER online analysis showed that risk factors and immune cells were significantly correlated. In conclusion, the risk model based on 10 ferroptosis regulators and markers has a good prognostic value for ovarian cancer patients.

## Introduction

Ovarian cancer is the deadliest gynecologic cancer due to its high rate of recurrence and limited early diagnosis [1], putting a huge cost upon patients and society. There has been a considerable decrease in the incidence and death rates of ovarian cancer over the last several decades because of advancements in therapy; however, the death rate from ovarian cancer remains high, and less than one-half of patients survive for more than five years [2]. Modern treatments for ovarian cancer patients vary with individual specialty, depending on histological type and cancer stage. In general, the standard of care for patients with ovarian cancer consists of primary debulking surgery and platinum-based combination chemotherapy [3]. Nevertheless, for certain patients, particularly those with recurring disorders, standard treatment alone is insufficient in the majority of cases.

Ferroptosis, a distinct form of programmed cell death, was first described in 2012 by Dixon [4]. Ferroptosis, in contrast to autophagy and apoptosis, is defined as an iron- and ROS (reactive oxygen species)-dependent cell death characterized primarily by cytological alterations, such as diminished or disappeared mitochondrial cristae, rupture of outer mitochondrial membrane [5–9]. These cellular abnormalities were caused by a loss of plasma membrane selective permeability as a result of severe membrane lipid peroxidation and the initiation of oxidative stress [10]. Ferroptosis process regulation involves various genes, which could be briefly grouped into drivers that drive ferroptosis, suppressors that suppress ferroptosis, and markers that indicate ferroptosis occurrence [11]. Accumulating evidence has indicated that ferroptosis is closely correlated with various diseases, including neurodegenerative disorders [12], ischemia/reperfusion damage [13, 14], acute renal injury [15], and malignancies, etc. Thus, ferroptosis regulators and markers might be potential targets and markers for diseases, including cancers.

A large body of data suggests the inhibitory effect of ferroptotic cell death on tumor development. Despite the fact that oxidative phosphorylation is most effective in the production of ATP, numerous tumor cells undergoing metabolic reprogramming primarily produce ATP from cytosolic aerobic glycolysis combined with lactate fermentation. This metabolic reprogramming in malignancies was notably found in the 1920s by Warburg and Cori and has been proposed as a tumor cell way to prevent toxic ROS levels [16, 17]. Nevertheless, maintaining this Warburg effect necessitates elevated glucose uptake and increased metabolic activity which make cancer cells significantly dependent on the anti-oxidant mechanism and possibly even more vulnerable to oxidative stress [18, 19]. As a result, highly proliferative tumor cells have been found to require handling of increased ROS levels in order to effectively develop tumors [20–22]. Thus, cancer cells have a much higher demand for iron than non-cancer cells and such reliance upon iron will make tumor cells more sensitive to ferroptosis. Cancer cells also have ROS tolerance and dependence on iron in metabolism, making them more susceptible to ferroptosis. Given these previous findings, ferroptosis regulators (drivers and suppressors) and markers might be promising and therapeutic targets for cancer. Regarding ovarian cancer, a potential link between ferroptosis and ovarian cancer is demonstrated based on previous studies [23, 24], and a focus on ferroptosis may provide a sophisticated therapeutic strategy for treating ovarian cancer [25]. Immunotherapy, including immune checkpoint inhibitors, has been used to treat ovarian cancer [26]. It is clinically important to investigate the factors affecting the prognosis of immunotherapy. Accumulating evidence suggests that ferroptosis plays a crucial role in immune evasion [26]. In turn, in immunotherapy, CD8 + T cells can trigger ferroptosis in cancer cells [27]. Targeting ferroptosis in combination with immunotherapy might become a prospective strategy for cancer therapy.

Although several studies have proposed a variety of prognostic models based on differentially expressed genes between ovarian cancer and para-cancerous samples, the sensitivity and specificity of these prediction models remain unsatisfactory. Recently, the Least Absolute Shrinkage and Selection Operator (LASSO) has been proposed as a regression algorithm for high-dimensional data [28], which was applied to select the most prominent predictive characteristics in the training dataset. Herein, cases of ovarian cancer patients with clinical information and expression profile information were obtained from The Cancer Genome Atlas Ovarian Cancer (TCGA-OV). Cases were calculated for immune scores using the Estimation of Stromal and Immune cells in Malignant Tumors using Expression data (ESTIMATE) algorithm [29], ferroptosis regulators and markers were obtained from the FerrDb database (http://www.zhounan.org/ferrdb/), and differentially expressed ferroptosis regulators and markers between high- and low-immune score groups were analyzed. Then, ferroptosis regulators and markers associated with ovarian cancer patients’ OS were identified using the univariate and multivariate Cox regression analyses. Next, The TCGA-OV cases were randomly divided into a training set and a validation set. The LASSO-regularized linear Cox regression was employed to construct a risk model consisting of ferroptosis regulators and markers. The risk model’s prognostic value was then validated using a time-dependent receiver-operating characteristic (ROC) curve analysis, multivariate Cox’s proportional hazard regression model analysis, and nomograms. The LASSO-regularized linear Cox regression was used to establish a ferroptosis regulator- and markers-based risk model for ovarian cancer prognosis.

## Materials and methods

### Source data of OV from TCGA and GEO database

All data were collected from the Cancer Genome Atlas (TCGA) database (https://tcga-data.nci.nih.gov/tcga/), including gene expression profiles and clinical data of patients enrolled. The inclusion criteria of the present study included: (1) Patients with complete gene expression profiles that could be applied to assess immune scores using the ESTIMATE algorithm; (2) patients with complete clinical data; and (3) patients with complete prognosis data. Then, a total of 378 cases of ovarian cancer patients with clinical information and expression profile information were obtained from TCGA-OV database.

The microarray data have been deposited in NCBI’s Gene Expression Omnibus and are accessible through Gene Expression Omnibus (GEO; https://www.ncbi.nlm.nih.gov/geo/) Series accession number. Gene expression data have been archived as microarray datasets in GEO repository at the NCBI archives and are accessible through GEO Series accession number GSE63885. The GSE63885 dataset includes the gene expression profiles from 101 ovarian carcinoma specimens: 73 serous, 12 endometrioid, 9 clear cells, and 7 undifferentiated.

### Data processing

A total of 378 cases of ovarian carcinoma patients with clinical information and expression profile information were obtained from The Cancer Genome Atlas Ovarian Cancer (TCGA-OV). Each patient’s immune score was evaluated by the ESTIMATE algorithm [29]. According to this score and using the mean score as a cut-off, ovarian cancer patients were assigned into high immune score (*N* = 185) and low immune score (*N* = 193) groups for Kaplan-Meier survival analysis. Subsequently, based on machine learning, these 378 patients with ovarian cancer were randomly divided into a training set (*N* = 189) and a validation set (*N* = 189). The clinical information is shown in Table 1.

**Table 1:** Clinical information of patients in the high-, low-immune score groups, training set, and validating set

Characteristics	Total (*N* = 378)		High (*N* = 185)		Low (*N* = 193)		Training (*N* = 189)		Valiating (*N* = 189)	
Age at diagnosis (y)	59(30–87)		58(34–87)		61(30–87)		60(30–85)		58(36–87)	
Overall Survival (m)	34.13(0.27–182.7)		35.3(0.3-155.5)		31.63(0.27–182.7)		32.97(0.27–155.5)		35.1(0.53–182.7)	
Stage										
I	1	0.26%	1	0.54%	0	0.00%	1	0.53%	0	0.00%
II	23	6.08%	14	7.57%	9	4.66%	11	5.82%	12	6.35%
III	294	77.78%	139	75.14%	155	80.31%	142	75.13%	152	80.42%
IV	57	15.08%	30	16.22%	27	13.99%	33	17.46%	24	12.70%
-	3	0.79%	1	0.54%	2	1.04%	2	1.06%	1	0.49%
Immune score (mean)	34.27 (-14.15-84.41)		50.21 (34.33–84.41)		19.00 (-14.15-34.13)		33.08 (-8.93-80.46)		35.46 (-14.15-84.41)	

### Ferroptosis regulators and markers

A total of 259 ferroptosis regulators and markers were obtained from the FerrDb database (http://www.zhounan.org/ferrdb/) [11], including 108 drivers, 69 suppressors, and 111 markers. Based on patients’ expression profiles in high immune scores (*N* = 185) and low immune score (*N* = 193) groups, ferroptosis regulators and markers were applied for differentiation analysis (∣log2FC∣>1, *P* < 0.05), identifying differentially expressed ferroptosis regulators and markers.

### Identification of prognostic signature

The LASSO-regularized linear Cox regression was implemented using the sklearn library in Python for the Lasso regression model. For univariate and multivariate Cox regression analyses, we utilized the survival package in the R language. Based on 189 samples of ovarian cancer patients in the training set, the prognostic value of differentially expressed ferroptosis regulators and markers was evaluated by the multivariate Cox regression analyses identifying ferroptosis regulators and markers associated with ovarian cancer patients’ overall survival. Then, the coefficients of these ferroptosis regulators and markers linked to ovarian cancer patients’ overall survival were analyzed using the LASSO-regularized linear Cox regression. The following formula was employed to calculate each patient’s risk score for prognostic signature: risk score = expression of gene_1_ × β_1_gene_1_ + expression of gene_2_ × β_2_gene_2_ + expression of gene_3_ × β_3_gene_3_ [30, 31]. For the present study, the formula is: Risk_score = Exp(LAMP2)*-1.0014834743436096 + Exp(NOS2)*2.829059192228839 + Exp(ALOX5)*6.378912927866197 + Exp(CD44)*-2.464480478133204 + Exp(CHMP5)*-0.08451646841520694 + Exp(FH)*-8.973630741722427 + Exp(GOT1)*7.001890615921591 + Exp(DUOX2)*8.898412094886682 + Exp(SLC7A11)*-10.956246534207127 + Exp(DDIT3)*-6.697215501726477. A 10-ferroptosis-related gene signature was obtained. The risk score for each patient in the training set, validation set, and independent dataset GSE63885 was calculated; patients in each dataset were assigned into high- or low-risk score groups, and the prognostic value of the risk model was evaluated using Kaplan-Meier survival analysis.

### Genomic-clinicopathologic nomogram

The nomogram was constructed based on the LASSO-regularized Cox regression model using the “survival” and “rms” package in the R language. The Consistency index (C index) was calculated using the survConcordance function in “Survival” package in R language, which is the capability of the model to distinguish between patients who survived and those who did not. The performance of the nomogram was evaluated using a bootstrap resampling approach with 1000 iterations to estimate the bias-corrected C-index. The bootstrap samples were generated by randomly sampling with replacement from the original dataset, using the same sample size as the original. This process was repeated 1000 times to ensure the stability and reliability of the nomogram performance estimates. Then, the calibration curves for the probability of 1-, 3-. and 5-year overall survival (OS) showed satisfactory agreement between predicted survival and actual observed survival.

### Sample collection

Ovarian cancer samples were collected from 20 ovarian carcinoma patients in Affiliated Tumor Hospital, Xinjiang Medical University. The inclusion criteria for ovarian cancer patients comprise: (1) diagnosed as serous ovarian cancer by postoperative pathology; (2) > 18 years old; (3) received ovarian cancer resection; (4) no chemotherapy, radiation therapy, immunotherapy, and other therapies prior to surgery; (5) no history of other malignancies or ovary-associated disorders. The normal ovarian tissue specimens were obtained from 10 cases who received myomectomy. All tissue samples were fixed in formalin until use. Written informed consent was obtained from all patients enrolled. Human tissue experiments were approved by the Ethics Committee of Affiliated Tumor Hospital, Xinjiang Medical University (approval ID: K-2021057). The clinicopathologic characteristics of 10 low-grade (pathological grade G1-G2) and 10 high-grade (pathological grade G3) ovarian carcinoma patients were included in Table 7.

### Immunohistochemistry (IHC)

The ovarian cancer tissue samples were embedded in paraffin. After deparaffinization and rehydration with graded alcohols, slices were rinsed twice with PBS for 10 min. Next, slices were incubated overnight with rabbit polyclonal primary antibody of CD1a (Cat# ab108309, 1/1000, Abcam, Cambridge, MA, USA), CD4 (Cat# ab133616, 1/500, Abcam), CHMP5 (Cat# ab96273, 1/500, Abcam) and DDIT3 (Cat# ab11419, 1/100, Abcam), followed by incubation at 37℃ for 30 min with 45µl secondary antibody horseradish peroxidase-conjugated goat polyclonal anti-rabbit or mouse IgG H&L (HRP) (Cat# ab6721 and ab205719 1:1000, Abcam). After staining with 3, 3’-diaminobenzidine (DAB) for 3 min, slices were rinsed with water for 10 min. After counterstaining with hematoxylin, slices were rinsed with water for 10 min, and then dehydrated and cleared. Lastly, a light microscope was employed to observe and photograph slices. IHC score was calculated by photographing at least 5 random fields of each specimen. We employed a combined score system based on both intensity and extent for calculating the IHC score. 1) Staining intensity is graded as 0, 1, 2, or 3 (for negative, weak, moderate, and strong). 2) The percentage of positive cells is scored as follows: 0, < 5%; 1, 6–25%; 2, 26–50%; 3, 51–75%; and 4, > 76%. The final IHC score is the product of two indicators.

### Statistical analyses

All statistical analysis uses Python (version 3.7.5; https://www.python.org/) and R language (version 4.0.2; https://www.r-project.org/). *P* < 0.05 is considered statistically significant. Delete the missing clinical data from the list; delete the entire sample from the analysis if the value of any parameter is missing. OS is defined as the time interval between the date of the first patient visit and the date of death. The mean value comparison of continuous variables uses a two-sided *t*-test. Kaplan-Meier method was used for survival analysis of high-risk group and low-risk group using a two-sided log-rank test in Python. The log-rank test, a non-parametric test used to compare the survival distributions of two groups, was employed to assess the significance between high and low-risk groups in the Kaplan-Meier survival analysis. This test is particularly suited for censored data and is widely used in survival analysis to test the null hypothesis that there is no difference in survival between the groups being compared. Patients with risk scores above the median were classified into the high-risk group, whereas those with scores below the median were classified into the low-risk group. We conducted two-sided *t*-tests for the hypothesis testing of 259 comparisons. This choice was made to account for the possibility of both positive and negative effects. FDR correction was performed with a cut-off value of 0.05 [32].

## Results

### Ferroptosis regulator and marker-based risk model established using the LASSO-regularized linear Cox regression

**Fig. 1:**
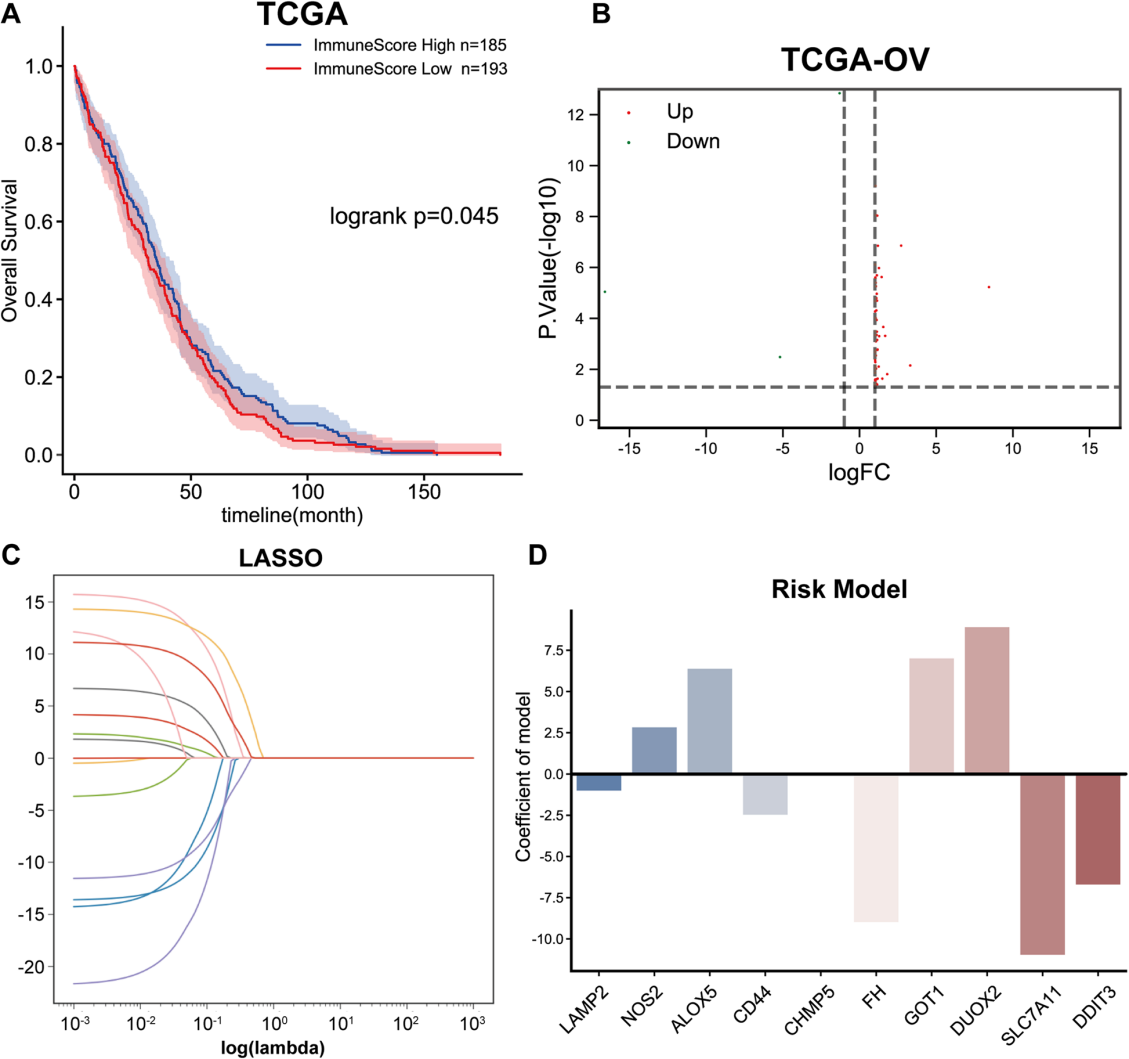
Ferroptosis regulator and marker-based risk model established using the Least Absolute Shrinkage and Selection Operator (LASSO)-regularized linear Cox regression. **A** A total of 378 patients were calculated for immune score by ESTIMATE algorithm and assigned into high- or low-immune score group; the association of immune score with the overall survival in patients with ovarian cancer was analyzed using a Kaplan-Meier estimate. **B** Ferroptosis regulators and markers based on FerrDb database (http://www.zhounan.org/ferrdb/) were analyzed for differential expression in high- or low-immune score groups. **C** The coefficients of differentially expressed ferroptosis regulators and markers in TCGA-OV training set (*n* = 189) were calculated by multivariate Cox regression using LASSO-regularized linear Cox regression

First, a total of 378 ovarian cancer subjects with clinical information and expression profile information from TCGA-OV were calculated for immune score with ESTIMATE algorithm and assigned into high- or low-immune score group (Table 1) using the mean immune score as the cut-off; a Kaplan-Meier estimate was employed to analyze the correlation between immune score and ovarian carcinoma patients’ OS. As shown by Fig. 1A, patients from the high-immune score group exhibited an obviously better prognosis.

 Then, the list of the ferroptosis-related regulators and markers was retrieved from the FerrDb database (http://www.zhounan.org/ferrdb/). A total of 259 ferroptosis-associated regulators and markers were analyzed for differential expression in the high- or low-immune score group and 64 were found with significant differences (|log_2_FC|> 1, *P* < 0.05) (Fig. 1B; Table 2). Based on 189 ovarian cancer samples in the training set, the prognostic value of 64 ferroptosis-related regulators and markers was evaluated by multivariate risk regression analysis, and 15 of them showed to be remarkably linked to ovarian carcinoma patients’ OS (*P* < 0.05) (Table 3).

**Table 2:** Differentially-expressed ferroptosis regulators in high- and low-immune score groups

Gene.symbol	log_2_FC	Regulation	*P*.Value	adj.*P*.Val
ZEB1	-1.303268835	Down	1.43E-13	1.70E-11
SAT1	1.040029699	Up	6.19E-10	3.67E-08
SQSTM1	1.052879177	Up	8.80E-09	3.13E-07
NQO1	1.152022282	Up	9.25E-09	3.13E-07
MYB	2.713284259	Up	1.39E-07	3.71E-06
CYBB	1.193947248	Up	1.41E-07	3.71E-06
FTH1	1.019080646	Up	7.74E-07	1.53E-05
ALOX5	1.265722731	Up	1.06E-06	1.89E-05
SOCS1	1.128552839	Up	2.04E-06	3.03E-05
NCF2	1.439163523	Up	2.39E-06	3.29E-05
RELA	1.025513027	Up	2.50E-06	3.29E-05
SCP2	1.041083389	Up	2.64E-06	3.30E-05
MUC1	1.055501129	Up	3.99E-06	4.50E-05
PML	1.066477531	Up	5.52E-06	5.95E-05
ZFP69B	8.44485485	Up	5.92E-06	6.10E-05
DPP4	-16.60461898	Down	9.07E-06	8.96E-05
ANGPTL7	1.121711507	Up	1.09E-05	1.03E-04
TAZ	1.096090796	Up	1.61E-05	1.41E-04
PCK2	1.135223683	Up	2.02E-05	1.65E-04
HSPB1	1.015436275	Up	2.12E-05	1.67E-04
CDKN2A	1.093951625	Up	4.88E-05	3.73E-04
PRDX1	1.014939856	Up	5.34E-05	3.96E-04
ATG3	1.047127674	Up	9.54E-05	6.46E-04
PHKG2	1.059605756	Up	1.07E-04	6.83E-04
CD44	1.112528197	Up	1.16E-04	7.25E-04
PTGS2	1.548795188	Up	2.18E-04	1.20E-03
CISD2	1.039490309	Up	3.23E-04	1.70E-03
STEAP3	1.127225037	Up	3.33E-04	1.72E-03
FH	1.024963136	Up	4.43E-04	2.11E-03
CHMP5	1.025211575	Up	4.48E-04	2.11E-03
MT1G	1.115455889	Up	4.50E-04	2.11E-03
DDIT3	1.026308285	Up	4.54E-04	2.11E-03
CDO1	1.673151129	Up	4.87E-04	2.22E-03
LINC00472	1.279821228	Up	5.01E-04	2.24E-03
AIFM2	1.147153689	Up	7.00E-04	3.07E-03
NOS2	1.067585893	Up	8.01E-04	3.45E-03
GCH1	1.170107442	Up	1.67E-03	6.81E-03
AKR1C1	1.16582017	Up	1.72E-03	6.91E-03
CISD1	1.038092873	Up	1.82E-03	7.06E-03
HERPUD1	1.024418643	Up	2.15E-03	7.96E-03
TFAP2C	1.063103058	Up	2.88E-03	1.02E-02
SLC7A11	-5.1864395	Down	3.31E-03	1.14E-02
GPX4	1.010620025	Up	4.05E-03	1.37E-02
CAPG	1.017779794	Up	4.61E-03	1.53E-02
LAMP2	1.019202163	Up	4.64E-03	1.53E-02
TNFAIP3	1.031849711	Up	5.24E-03	1.70E-02
NOX4	3.306540785	Up	7.09E-03	2.24E-02
GABARAPL2	1.033479799	Up	7.56E-03	2.36E-02
AKR1C2	1.248168752	Up	7.84E-03	2.41E-02
HMOX1	1.08061761	Up	9.33E-03	2.78E-02
TXNIP	1.037383732	Up	9.40E-03	2.78E-02
PEBP1	1.013710687	Up	1.02E-02	2.95E-02
MAPK3	1.016838332	Up	1.37E-02	3.68E-02
IL6	1.802622241	Up	1.56E-02	4.14E-02
ATP6V1G2	1.487442492	Up	2.32E-02	5.92E-02
DUOX2	1.176234804	Up	2.33E-02	5.92E-02
PROM2	1.09987205	Up	2.45E-02	6.11E-02
YY1AP1	1.026722519	Up	2.50E-02	6.18E-02
UBC	1.009911407	Up	2.65E-02	6.40E-02
BID	1.033659428	Up	2.96E-02	7.01E-02
ATG7	1.08770804	Up	3.66E-02	8.43E-02
TF	1.149335695	Up	4.00E-02	9.11E-02
GOT1	1.024804256	Up	4.12E-02	9.21E-02
STAT3	1.011819434	Up	4.64E-02	1.02E-01

**Table 3:** Ferroptosis regulators associated with ovarian cancer patients’ overall survival

Gene	coef	exp(coef)	se(coef)	z	p
FH	-1.633849	0.195177	0.35938	-4.546	5.46E-06
CD44	-0.962617	0.381892	0.231665	-4.155	3.25E-05
SLC7A11	-0.81054	0.444618	0.212607	-3.812	1.38E-04
ANGPTL7	-6.126365	0.002185	1.96875	-3.112	1.86E-03
TAZ	-0.999228	0.368164	0.346508	-2.884	3.93E-03
DUOX2	1.656473	5.240794	0.578878	2.862	4.22E-03
GABARAPL2	0.912891	2.491515	0.335007	2.725	6.43E-03
NOS2	1.787064	5.971895	0.707606	2.526	1.16E-02
LAMP2	-0.70243	0.49538	0.291433	-2.41	1.59E-02
ALOX5	0.604862	1.830999	0.255935	2.363	1.81E-02
CHMP5	-0.736627	0.478726	0.322767	-2.282	2.25E-02
ZEB1	0.744825	2.106073	0.326816	2.279	2.27E-02
GOT1	0.70303	2.019863	0.317499	2.214	2.68E-02
DDIT3	-0.572935	0.563868	0.263155	-2.177	2.95E-02
PHKG2	0.941572	2.56401	0.473549	1.988	4.68E-02

Subsequently, 378 ovarian cancer patients from TCGA-OV were randomly separated into a training set (*N* = 189) and a validation set (*N* = 189) (Table 1). For predicting the clinical outcome of these ferroptosis-related regulators and markers, a LASSO-regularized linear Cox regression analysis was carried out to establish the risk score model based on 189 samples from the training set. According to the minimum criteria, a 10-gene risk signature was established (Fig. 1C). The 10 ferroptosis-related regulators and markers are: LAMP2, NOS2, ALOX5, CD44, CHMP5, FH, GOT1, DUOX2, SLC7A11, and DDIT3.

### The prognostic value of the risk score model

**Fig. 2:**
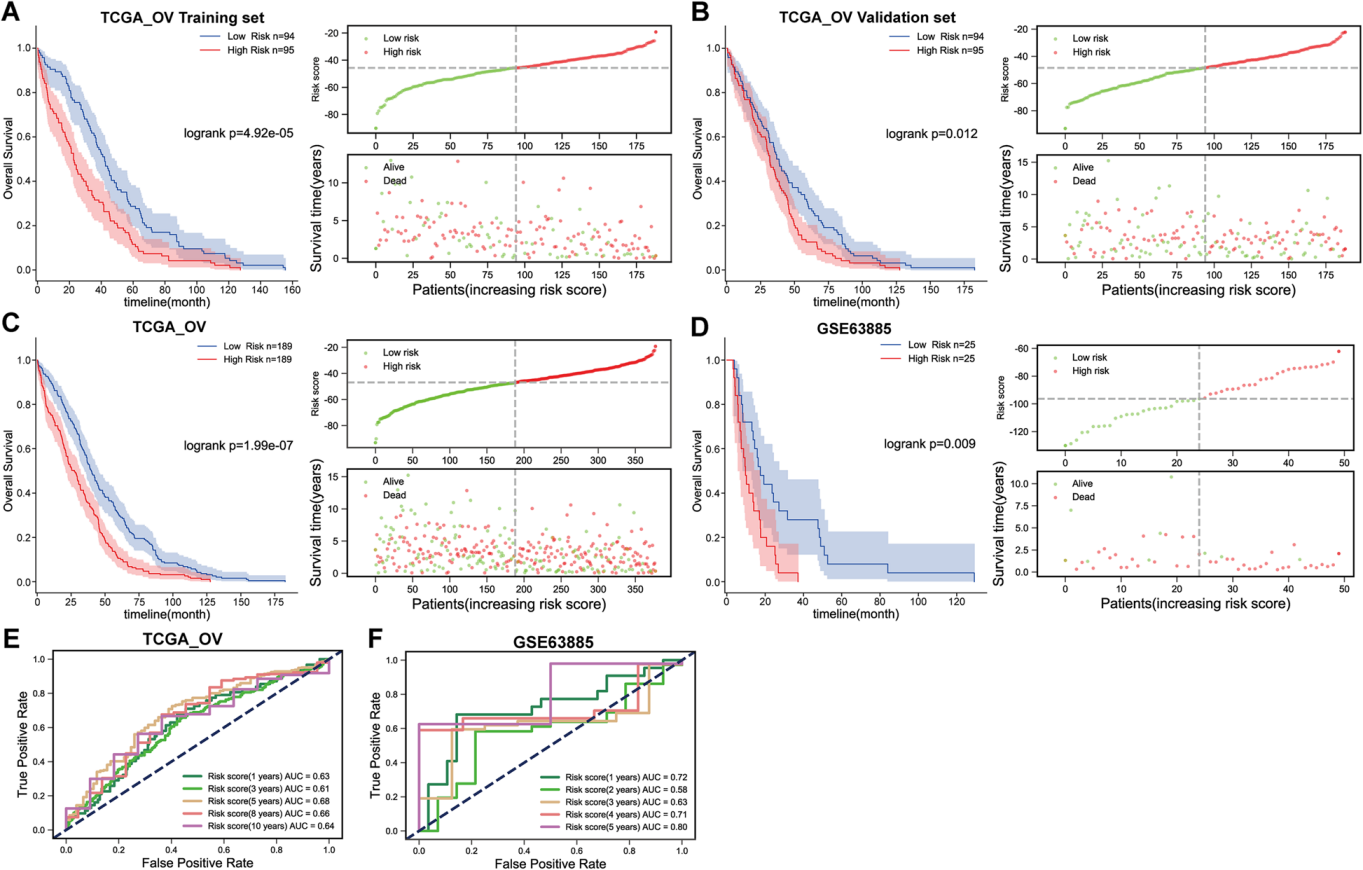
The prognostic value of the risk score model. **A** Risk scores of cases in TCGA-OV (training set, *N* = 189) were calculated using the formula described in the M&M section. Cases were divided into two groups using the median value of the risk score as a cut-off; the correlation of the subjects’ overall survival with risk score was analyzed. **B** Risk scores of cases in TCGA-OV (validation set, *N* = 189) were calculated using formula described in the M&M section. Cases were divided into two groups using the median value of the risk score as a cut-off; the correlation of the subjects’ overall survival with risk score was analyzed. **C** Risk scores of cases in TCGA-OV (all subjects, *N* = 378) were calculated using formula described in the M&M section. Cases were divided into two groups using the median value of the risk score as a cut-off; the correlation of the subjects’ overall survival with risk score was analyzed. **D** Risk scores of cases in GSE63885 were calculated using formula described in the M&M section. Cases were divided into two groups using the median value of the risk score as a cut-off; the correlation of the subjects’ overall survival with risk score was analyzed. **E** Receiver operating characteristic (ROC) curves showed the predictive efficiency of the risk score model in 1-, 3-, 5-, 8-, or 10-year overall survival based on cases from TCGA-OV. **F** ROC curves showed the predictive efficiency of the risk score model in 1-, 2-, 3-, 4-, or 5-year overall survival based on cases from GSE63885

According to the regression coefficient, the risk score for each subject in the training set, validation set, and independent dataset GSE63885 was calculated with the aforementioned formula. Subjects in each dataset were then assigned into high- and low-risk score groups based on the median score in each group. In the TCGA-OV training set (Fig. 2A), TCGA-OV validation set (Fig. 2B) and TCGA-OV entire set (Fig. 2C), and independent dataset GSE63885 (Fig. 2D), the subjects with lower risk scores obtained better overall survival. For further confirming the prognostic value of the risk score model, receiver operating characteristic (ROC) curves [33] were drawn, and the predictive efficiency of the risk score model in 1-, 3-, 5-, 8-, or 10-year overall survival based on cases from TCGA-OV was shown. As shown by Fig. 2E. the area under the curves (AUC) for OS from TCGA-OV were 0.63 (1 year), 0.61 (3 year), 0.68 (5 year), 0.66 (8 year), and 0.64 (10 year). Similarly, ROC curves showed the predictive efficiency of the risk score model in 1-, 2-, 3-, 4-, or 5-year overall survival based on cases from GSE63885. As shown by Fig. 2F, the AUC for OS from GSE63885 were 0.72 (1 year), 0.58 (2 year), 0.63 (3 year), 0.71 (4 year), and 0.80 (5 year). As revealed by the ROC curve, the risk score-based curve showed satisfactory predictive efficiency.

### Univariate and multivariate Cox analyses of clinicopathological variables

**Fig. 3:**
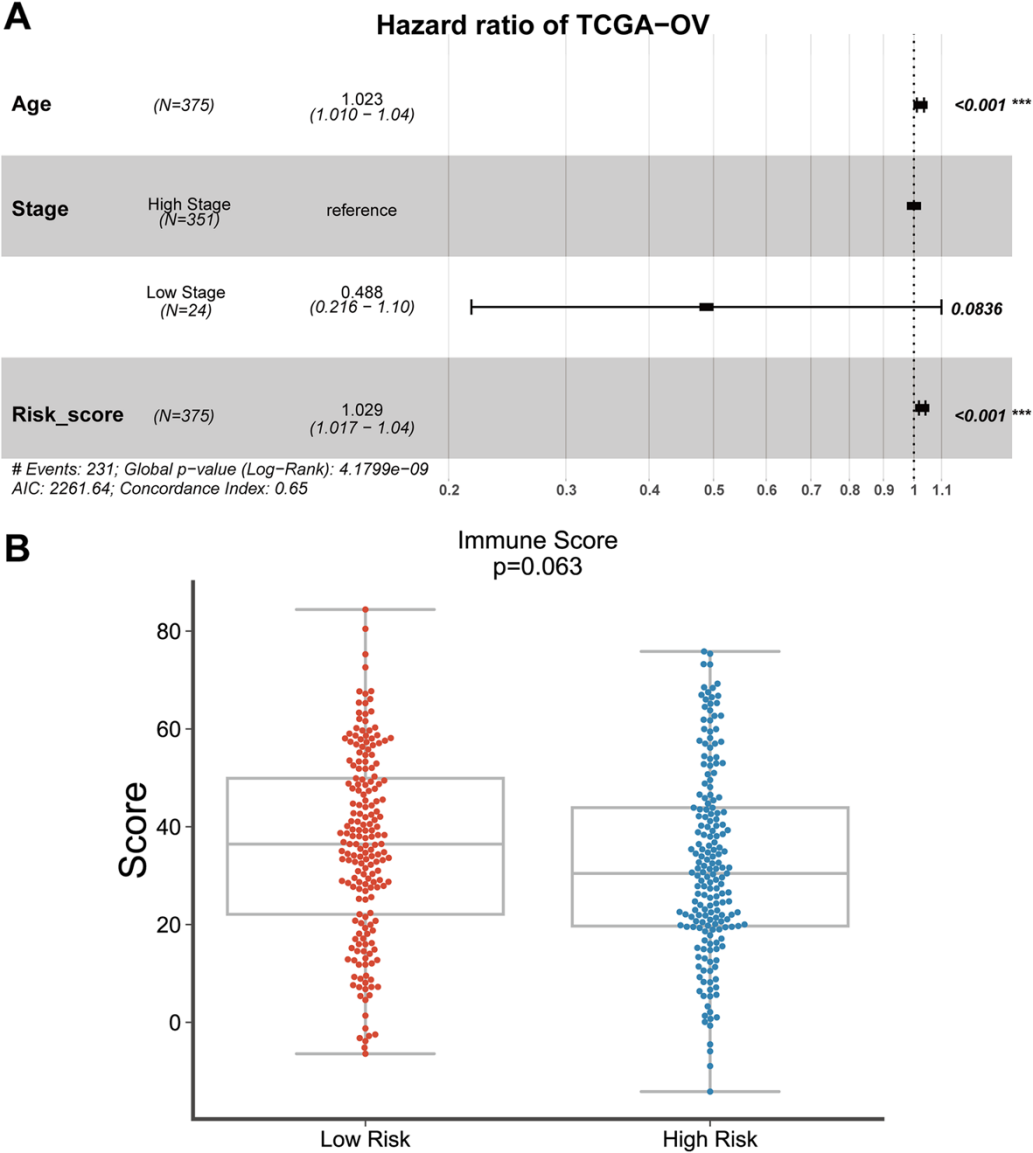
Cox multivariate analyses of clinicopathological variables. **A** The multivariate Cox regression analyzing the association between ovarian cancer patients’ overall survival and age, stage, and risk score based on TCGA-OV data. **B** Patients from TCGA-OV were assigned into high-risk or low-risk group and the immune scores were shown

Next, the clinical characteristics in TCGA-OV patients were analyzed using a univariate and multivariate Cox’s proportional hazard regression model (Table 4). Risk scores were considerably correlated with OS in OA patients. Also, Fig. 3A showed that according to TCGA-OV data, age (*P* < 0.001, HR = 1.023; 95% CI = 1.010–1.04) and the risk score (*P* < 0.001, HR = 1.029; 95% CI = 1.017–1.04) could predict patients’ OS. Moreover, patients from TCGA-OV were assigned into high-risk score or low-risk score group and the immune scores were shown. Figure 3B showed that patients in the high-risk score group obtained lower immune scores.

**Table 4:** Cox risk regression analysis on the correlation between the overall survival of ovarian cancer patients and clinical features

	Univariate		Multivariate	
	HR(95%CI)	*p*.value	HR(95%CI)	*p*.value
Age	1(1–1)	**5.20E-04**	1.02(1.01–1.04)	**4.35E-04**
Stage	0.47(0.21–1.1)	6.70E-02	0.49(0.22–1.10)	8.36E-02
Risk_score	1(1–1)	**6.30E-07**	1.03(1.02–1.04)	**5.93E-07**

**Fig. 4:**
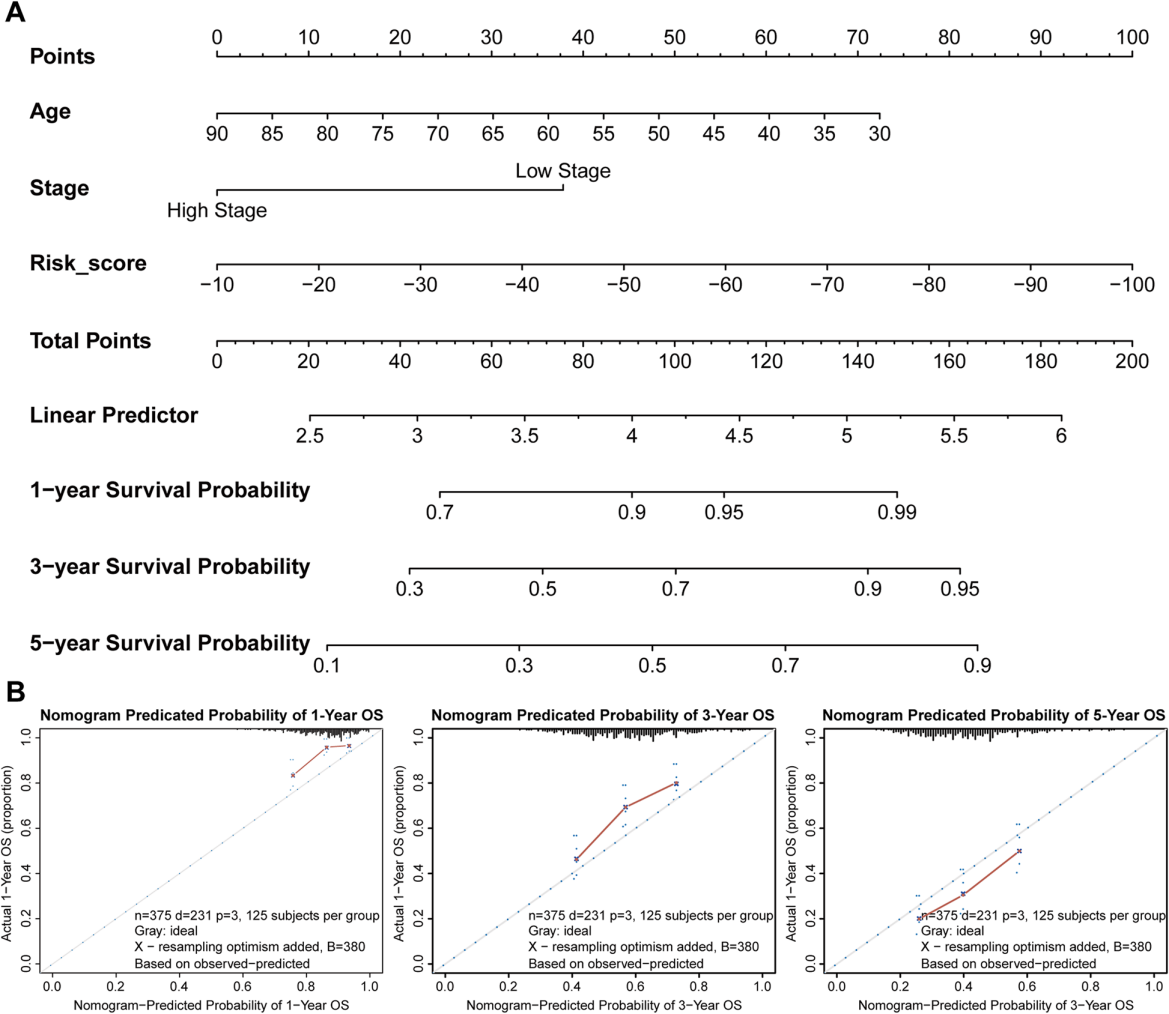
Nomogram analysis based on TCGA-OV. **A** Nomogram composed of age, stage, and risk score for the prediction of 1-, 3-, and 5-years survival probability. **B**-**D** Calibration plot for the evaluation of the nomogram in predicting 1-year, 3-years, and 5-years overall survival

For validating the prognostic value of the risk score model, a Nomogram analysis was performed based on TCGA-OV. Figure 4A-D showed that based on the cases with prognostic information from TCGA-OV, a prognostic nomogram predicting the 1-, 3-, and 5-year survival probability was established, respectively. The nomogram included age, stage, and the risk score. The C-index of the risk score was 0.65, suggesting a favorable prognosis of the model.

### Gene Ontology (GO) analysis on the 10 ferroptosis regulators and markers

**Fig. 5:**
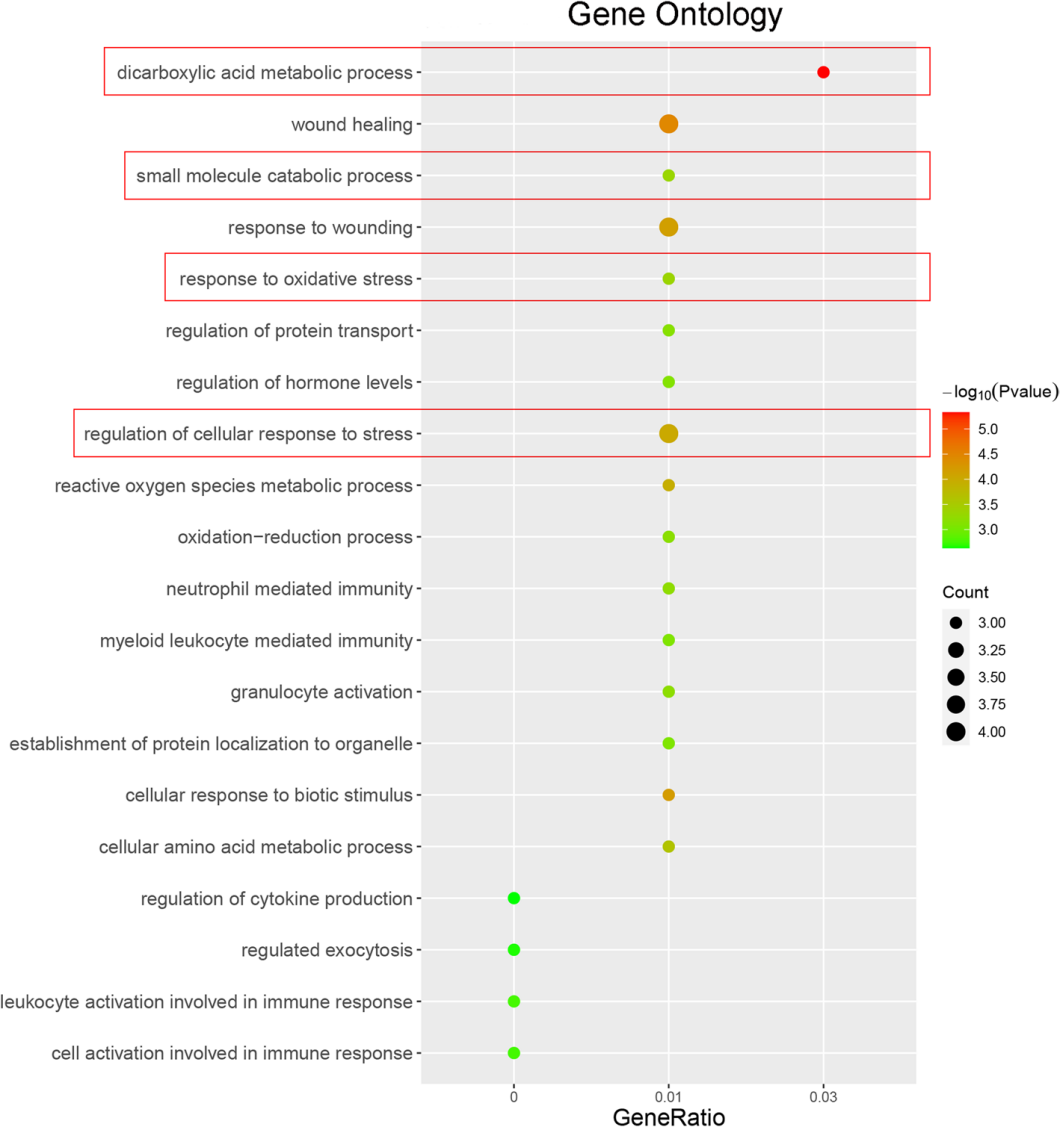
Gene Ontology (GO) analysis on the 10 ferroptosis regulators and markers

For an in-depth understanding of the 10 genes forming the risk model, GO analysis was conducted. Metascape online tool (https://metascape.org) [34] confirmed again the involvement of these 10 genes in ferroptosis such as cell metabolism, oxidation-reduction reactions, and oxidative stress, as well as immune activities such as leukocyte activation, granulocyte activation, and neutrophil-mediated immunity (Fig. 5; Table 5).

**Table 5:** Gene ontology functional enrichment annotation

Term	Pathway	*P*value	Count	GeneRatio
GO:0006520	cellular amino acid metabolic process	2.43E-04	3	0.01
GO:0043648	dicarboxylic acid metabolic process	4.69E-06	3	0.03
GO:0055114	oxidation-reduction process	6.54E-04	3	0.01
GO:0072593	reactive oxygen species metabolic process	1.14E-04	3	0.01
GO:0010817	regulation of hormone levels	7.55E-04	3	0.01
GO:0051223	regulation of protein transport	7.07E-04	3	0.01
GO:0080135	regulation of cellular response to stress	9.79E-05	4	0.01
GO:0006979	response to oxidative stress	4.52E-04	3	0.01
GO:0009611	response to wounding	7.12E-05	4	0.01
GO:0042060	wound healing	3.56E-05	4	0.01
GO:0044282	small molecule catabolic process	4.72E-04	3	0.01
GO:0072594	establishment of protein localization to organelle	8.35E-04	3	0.01
GO:0001817	regulation of cytokine production	2.33E-03	3	0
GO:0071216	cellular response to biotic stimulus	6.45E-05	3	0.01
GO:0002263	cell activation involved in immune response	1.78E-03	3	0
GO:0036230	granulocyte activation	6.32E-04	3	0.01
GO:0002366	leukocyte activation involved in immune response	1.75E-03	3	0
GO:0002444	myeloid leukocyte mediated immunity	8.13E-04	3	0.01
GO:0002446	neutrophil mediated immunity	6.10E-04	3	0.01
GO:0045055	regulated exocytosis	2.20E-03	3	0

**Fig. 6:**
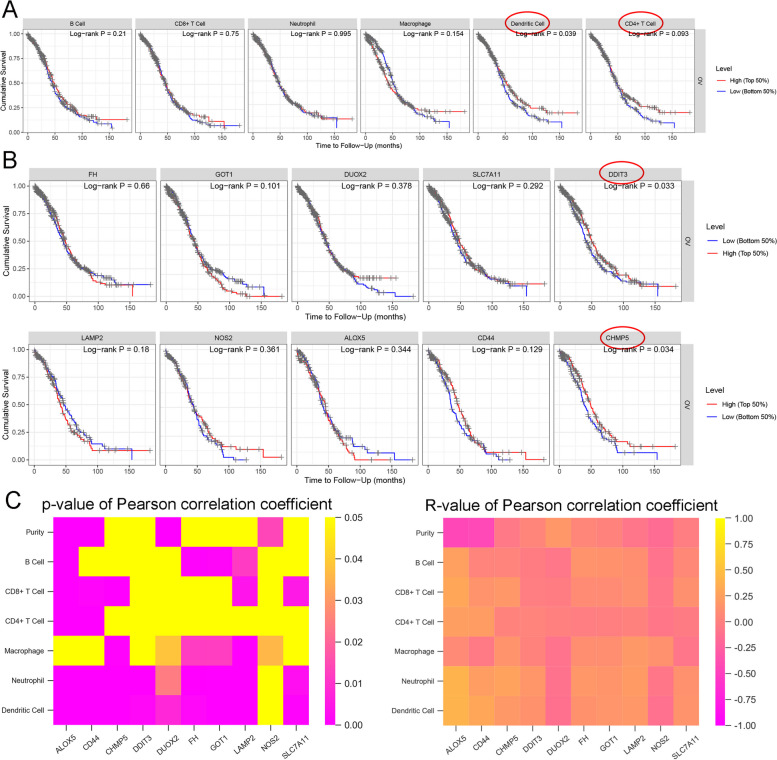
Association of 10 risk factors with immune cells in ovarian cancer. **A** The association of immune cells with ovarian cancer patients’ cumulative survival was analyzed using a Kaplan-Meier estimate. **B** The association of each of the 10 risk factors with ovarian cancer patients’ cumulative survival was analyzed using a Kaplan-Meier estimate. **C** The correlation between immune cells and the risk factors was analyzed using Pearson correlation coefficient. The left panel: heatmap showing the significance of the correlations (*P*-value); the right panel: heatmap showing the strength and direction of the correlations (*R*-value)

Since the GO analysis confirmed again that these 10 genes were enriched in ferroptosis biological activities and immune activities, next, the association of 10 risk factors with immune cells was analyzed in ovarian cancer. Through TIMER online analysis, the association of immune cells with ovarian cancer patients’ cumulative survival was analyzed using a Kaplan-Meier estimate; Fig. 6A showed that patients with higher dendritic cell or CD4 + T cell obtained better cumulative survival. Similarly, patients with higher DDIT3 or CHMP5 obtained better cumulative survival (Fig. 6B). Pearson correlation coefficient analysis was performed to analyze the correlation between immune cells and the risk factors; Fig. 6C; Table 6 showed that risk factors and immune cells were significantly correlated.

**Table 6:** Association of 10 risk factors with immune cells in ovarian cancer

	coef	HR	95%CI_l	95%CI_u	*p*.value	sig
B_cell	1.481	4.398	0.001	1.78E + 04	0.727	
CD8_Tcell	-4.513	0.011	0	2.35E + 00	0.099	·
CD4_Tcell	-17.009	0	0	1.00E-03	0.001	**
Macrophage	10.09	24095.888	4.037	1.44E + 08	0.023	*
Neutrophil	13.885	1071683.28	4.104	2.80E + 11	0.029	*
Dendritic	-0.558	0.573	0	7.47E + 02	0.879	
LAMP2	0.36	1.434	1.064	1.93E + 00	0.018	*
NOS2	-0.027	0.974	0.456	2.08E + 00	0.945	
ALOX5	0.051	1.052	0.872	1.27E + 00	0.595	
CD44	-0.208	0.812	0.662	9.97E-01	0.047	*
CHMP5	-0.262	0.769	0.581	1.02E + 00	0.067	·
FH	-0.697	0.498	0.365	6.80E-01	0	***
GOT1	0.447	1.563	1.2	2.04E + 00	0.001	**
DUOX2	0.28	1.323	0.92	1.90E + 00	0.131	
SLC7A11	-0.092	0.912	0.758	1.10E + 00	0.327	
DDIT3	-0.45	0.637	0.5	8.13E-01	0	***

* *p* < 0.05, ** *p* < 0.01, *** *p* < 0.005

### Expression of immune microenvironment and ferroptosis markers in ovarian cancer and its relationship with prognosis of ovarian cancer patients

**Fig. 7:**
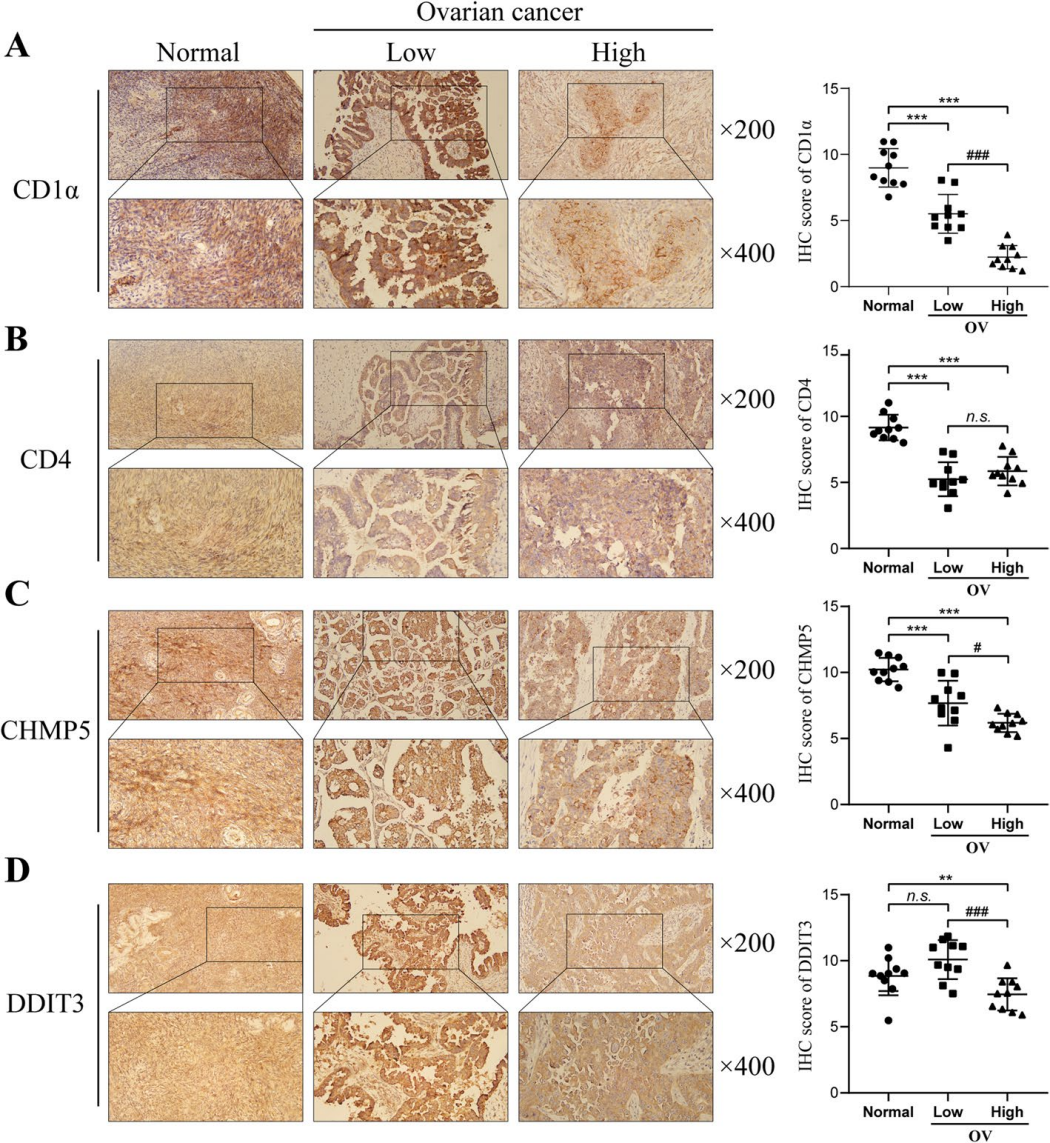
Expression of immune microenvironment markers (dendritic cell CD CD1α, CD4 T cell, CD4) and ferroptosis markers (CHMP5 and DDIT3) in ovarian cancer. IHC staining was applied to investigate the expression of CD1α (**A**), CD4 (**B**), CHMP5 (**C**) and DDIT3 (**D**) in normal ovarian tissues and low- or high-grade ovarian cancer tissues. **P < 0.01, ***P < 0.001 compared to normal ovarian tissues; #*P* < 0.05, ###*P* < 0.001 compared to low ovarian cancer tissues

To validate the bioinformatics analysis results, IHC staining was applied to investigate the expression of immune microenvironment markers (dendritic cell CD CD1α, CD4 T cell, CD4) and ferroptosis markers (CHMP5 and DDIT3) in normal ovarian tissues and low- or high-grade ovarian cancer tissues (Fig. 7). As showed in Fig. 7A-B, compared to normal ovarian tissues, CD1α and CD4 protein was markedly low-expressed in low- or high-grade ovarian cancer tissues; and CD1α was even lower in high-grade ovarian cancer tissues. For ferroptosis markers (CHMP5 and DDIT3), CHMP5 (Fig. 7C) and DDIT3 (Fig. 7D) protein expressions were notably down-regulated in high-grade ovarian cancer tissues. The clinicopathologic characteristics of clinical samples were shown in Table 7.

**Table 7:** The clinicopathologic characteristics of normal control and ovarian cancer patients

Case No.	Age	Histological subtype	Pathological grade	FIGO stage	Tumor location
N1	46	/	/	/	/
N2	53	/	/	/	/
N3	47	/	/	/	/
N4	50	/	/	/	/
N5	53	/	/	/	/
N6	49	/	/	/	/
N7	59	/	/	/	/
N8	48	/	/	/	/
N9	52	/	/	/	/
N10	49	/	/	/	/
P1	46	serous	G1-G2	II	bilateral
P2	30	serous	G1-G2	III	left
P3	34	serous	G1-G2	I	left
P4	55	serous	G1-G2	I	bilateral
P5	72	serous	G1-G2	III	left
P6	24	serous	G1-G2	I	right
P7	72	serous	G1-G2	III	left
P8	45	serous	G1-G2	III	bilateral
P9	28	serous	G1-G2	III	bilateral
P10	26	serous	G1-G2	II	bilateral
P11	44	serous	G3	III	bilateral
P12	54	serous	G3	III	right
P13	47	serous	G3	III	bilateral
P14	60	serous	G3	III	bilateral
P15	52	serous	G3	III	bilateral
P16	44	serous	G3	IV	left
P17	50	serous	G3	III	bilateral
P18	54	serous	G3	III	bilateral
P19	48	serous	G3	III	bilateral
P20	60	serous	G3	III	bilateral

*N* Normal, *P* Ovarian cancer patient, *FIGO* International Federation of Gynecology and Obstetrics

## Discussion

Herein, subjects from TCGA-OV were calculated for immune scores using the ESTIMATE algorithm and assigned into high- (*N* = 185) or low-immune (*N* = 193) score group; 259 ferroptosis regulators and markers were analyzed for expression and 64 were significantly differentially expressed between two groups. These 64 differentially expressed genes were applied for LASSO-regularized linear Cox regression for establishing ferroptosis regulators- and markers-based risk model, and a 10-gene signature was established. The ROC curve indicated that the risk score-based curve showed satisfactory predictive efficiency. Based on univariate and multivariate Cox risk regression analyses, age, and risk score were risk factors for ovarian cancer patients’ OS; patients in the high-risk score group obtained lower immune scores. The Nomogram analysis indicated that the prognostic outcomes of the model were consistent with the actual outcomes. GO functional enrichment annotation confirmed again the involvement of these 10 genes in ferroptosis and immune activities. Pearson’s correlation analysis showed that risk factors and immune cells were significantly correlated.

A growing body of data suggests that innate and adaptive immune systems play a critical role in the occurrence and progression of malignancies [35–37]. Furthermore, cancer immunotherapy has progressed so rapidly that immune-based ovarian cancer prognostic signatures might provide potential value for identifying new molecular targets [38]. By using the ESTIMATE algorithm, cases from TCGA-OV were calculated for immune scores and assigned into high- and low-immune score groups, and a Kaplan-Meier estimate then indicated that patients with higher immune scores obtained better overall survival, suggesting the prognostic value of the immune score. Given the crucial role of ferroptosis in ovarian cancer [39], 259 ferroptosis regulators and markers were obtained from the FerrDb database (http://www.zhounan.org/ferrdb/) [11] and differentially expressed ferroptosis regulators and markers between the immune score high group and low group were evaluated. Between the two groups, a total of 64 ferroptosis regulators and markers were differentially expressed, suggesting those ferroptosis-related genes are associated with the level of infiltrating of stromal and immune cells in ovarian cancer and might consist of a signature for ovarian cancer prognosis. Consistent with the hypothesis, interferon-γ has been reported to induce ferroptosis within tumor cells [40]. Moreover, the early ferroptotic cancer cells might significantly promote immune responses [41].

Exploiting ferroptosis inducers provides a potential therapeutic method for the treatment of ovarian cancer. Multiple conventional drugs might trigger ferroptosis in tumor cells, including Sulfasalazine, Artesunate, Temozolomide, and Cisplatin [42]. Similarly, ferroptosis suppressors represent promising therapeutic targets for treating ovarian cancer. A combination of Ferroptosis inducers combined with chemotherapeutic agents gains a remarkable synergistic effect on their anti-tumor activity [43]. In the present study, 15 of the 64 ferroptosis regulators and markers were considerably correlated with ovarian carcinoma patients’ OS. Furthermore, the LASSO-regularized linear Cox regression established a 10-gene risk model predicting ovarian cancer prognosis, consisting of LAMP2, NOS2, ALOX5, CD44, CHMP5, FH, GOT1, DUOX2, SLC7A11, and DDIT3, all of which could play a role in ovarian cancer development. For instance, cardamonin inhibited the mTOR lysosomal colocalization LAMP2, a well-known protein found in the membrane of lysosomes, decreasing Raptor siRNA SKOV3 cell proliferation [44]. NOS2 has initially been reported to play a significant anti-tumor role in the immune response; however, growing evidence has demonstrated that the expression level of NOS2 in tumor cells is usually associated with impaired prognosis [45]. Inherited variation in ALOX5 seems to affect ovarian cancer risk [46]. Exosomal CD44 has been reported to enhance the capacity of ovarian cancer cells to invade via CD44 transfer to the peritoneal mesothelium [47]. GOT1 modulates cellular metabolism by coordinating the use of carbohydrates and amino acids to satisfy dietary needs for long-term proliferation; in ovarian cancer, adapalene suppresses the growth of ovarian cancer cells by binding to GOT1 [48]. PARP pharmacologic inhibition represents a major factor in treating ovarian cancer with mutations in the BRCA; inhibition of PARP enhances ferroptosis through suppressing SLC7A11 and synergizes with ferroptosis inducers within BRCA-proficient ovarian cancer [49].

By grouping TCGA-OV training set and validation set and GSE63885 samples into 2 sub-groups with the median value of risk score as a cut-off, the Kaplan-Meier survival estimate indicated that the risk score was strongly associated with the overall survival of patients, the ROC curve demonstrated that the risk score-based curve showed satisfactory prediction efficiency, and the multivariate Cox’s proportional hazard regression analysis identified the risk score as an independent risk factor. More importantly, the intuitive and effective nomogram integrating age, stage, and risk model was established, and it could be used to predict the outcomes of patients quickly. Through TIMER online analysis and Kaplan-Meier analysis, patients with higher levels of dendritic cell, CD4 + T cell, DDIT3, and CHMP5 were associated with better cumulative survival. The clinical samples also confirmed that the levels of immune microenvironment markers (CD1α and CD4) and ferroptosis markers (CHMP5 and DDIT3) were lower in high-grade ovarian cancer tissues. These mRNAs might be used as therapeutic targets in treating ovarian cancer.

Regarding the limits associated with this research, firstly, the biological effect of the 10 identified genes should be verified by in vitro and in vivo experiments; considering the 10 genes were ferroptosis regulators and markers differentially expressed between the high-immune score group and low-immune score group, their functions upon ovarian cancer cell immune response and ferroptosis should be investigated in detail. Secondly, the risk score model needs to be further validated in several cohorts and large-size samples to assess the model’s generalizability.

Taken together, it is believed that the risk model based on 10 ferroptosis regulators and markers has a good prognostic value for ovarian cancer patients. It is worth noting that the risk score can also significantly distinguish ovarian cancer from normal samples, which may have a certain auxiliary value for early clinical screening of ovarian cancer.

## Data Availability

The datasets analyzed during the current study are available in the TCGA-OV repository (https://portal.gdc.cancer.gov/projects/TCGA-OV), GSE63885 repository (https://www.ncbi.nlm.nih.gov/geo/query/acc.cgi?acc=GSE63885) and FerrDb repository (http://www.zhounan.org/ferrdb/).
